# Supplementary material for: Hydrogen substituted graphdiyne as carbon-rich flexible electrode for lithium and sodium ion batteries
Source: Nat Commun. 2017 Oct 27;8:1172. doi: 10.1038/s41467-017-01202-2 (PMC5660080; doi:10.1038/s41467-017-01202-2)
Supplement: Supplementary file 1 — Supplementary Information [file 41467_2017_1202_MOESM1_ESM.pdf]

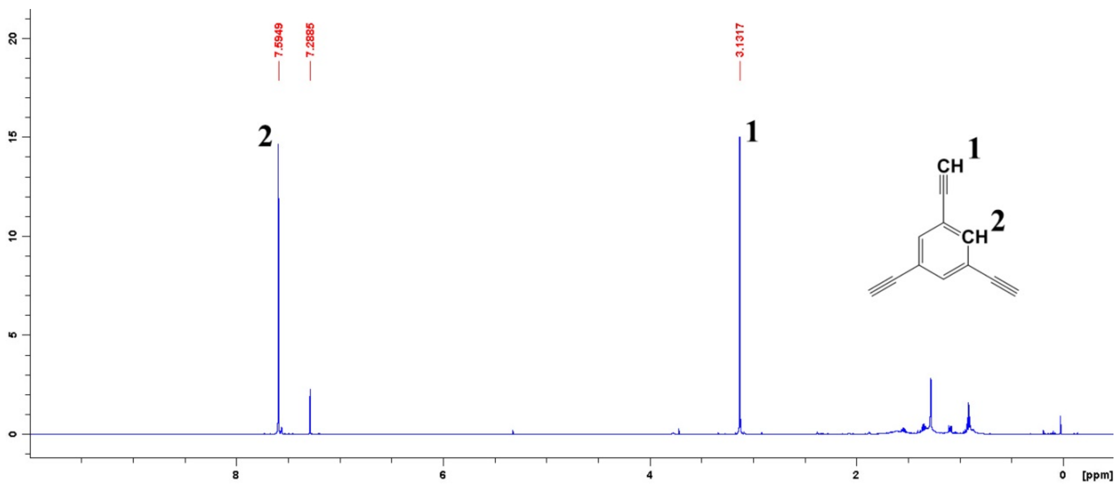

Supplementary Figure 1. ^{1}H NMR spectrum of Triethynylbenzene monomer.

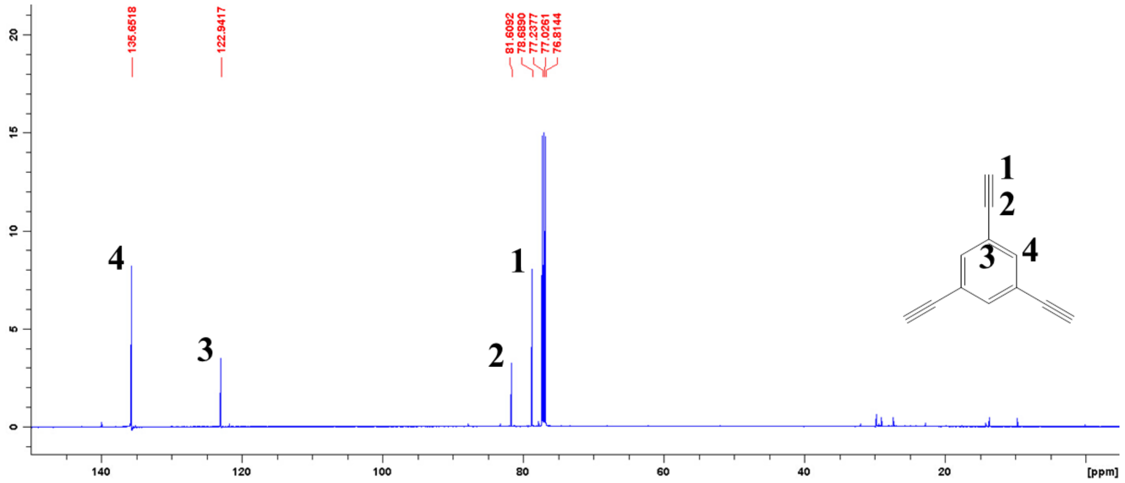

Supplementary Figure 2. ^{13}C NMR spectrum of Triethynylbenzene monomer.

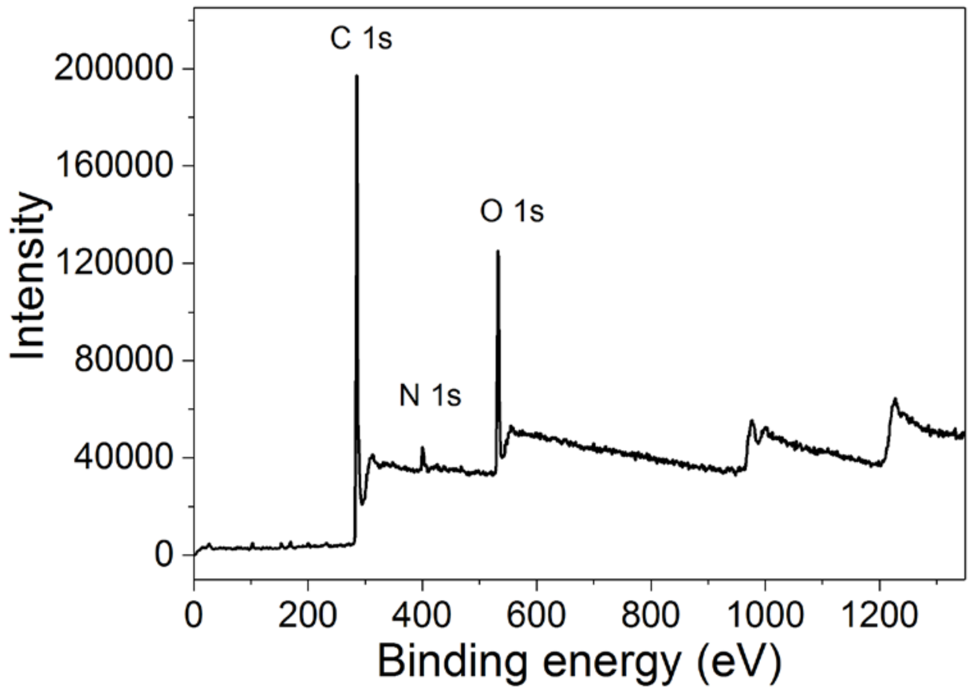

Supplementary Figure 3. XPS spectrum of HsGDY.

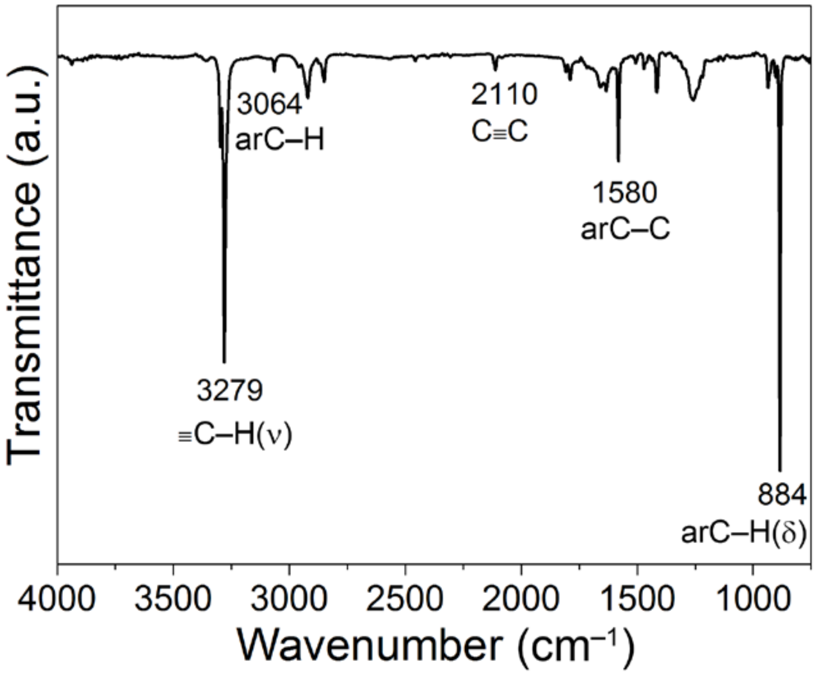

Supplementary Figure 4. FT-IR spectrum of Triethynylbenzene monomer.

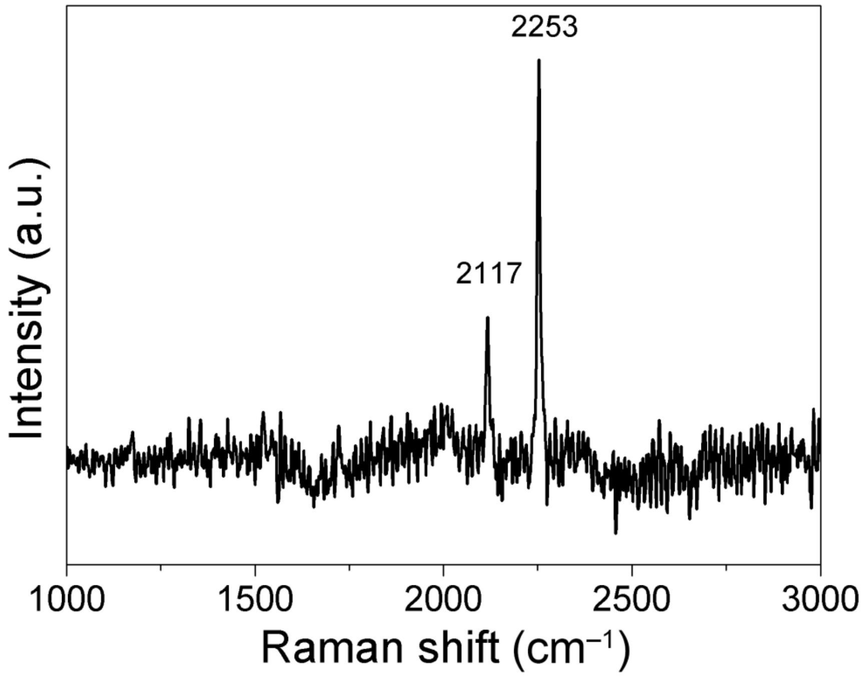

Supplementary Figure 5. Raman spectrum of Triethynylbenzene monomer.

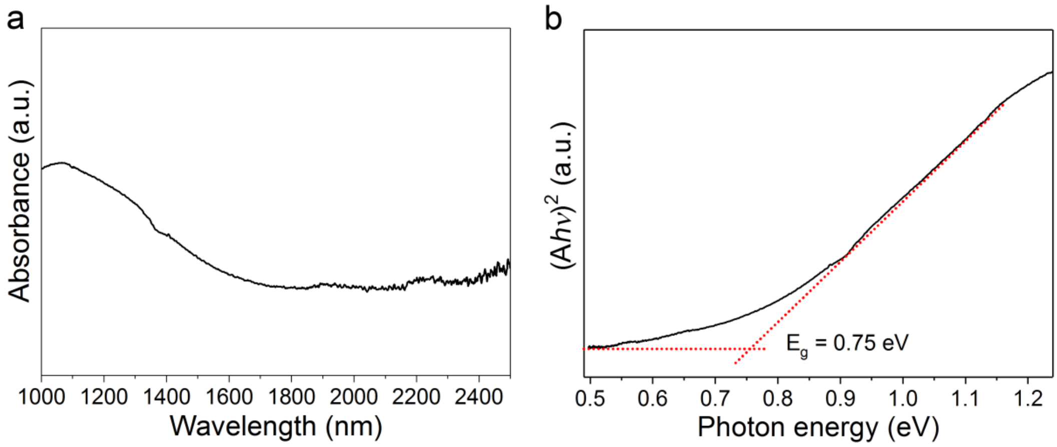

Supplementary Figure 6. Energy gap of HsGDY. (a) UV-vis spectrum of HsGDY. And (b) plots of $(Ah\nu)^2$ versus photon energy ($h\nu$).

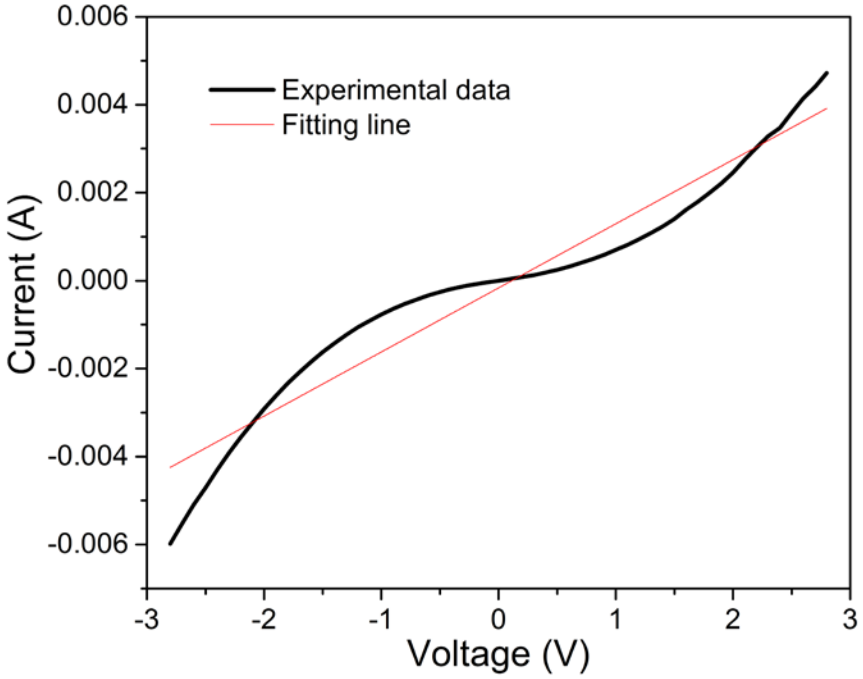

Supplementary Figure 7. I–V curves of the HsGDY film.

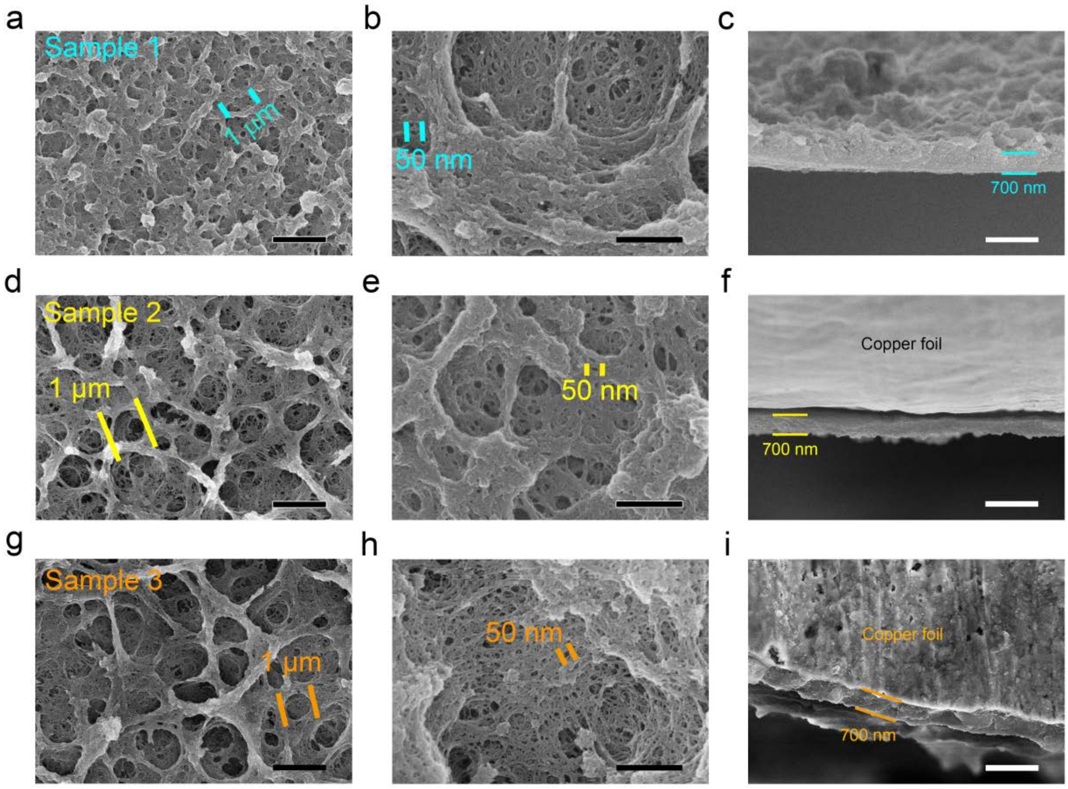

Supplementary Figure 8. The reproducibility of the HsGDY film. (a–c) sample 1, (d–f) sample 2, (g–i) sample 3. Scale bar, 2 μm (**a**, **c**, **d**, **f**, **g**, **i**), 500 nm (**b**, **e**, **h**).

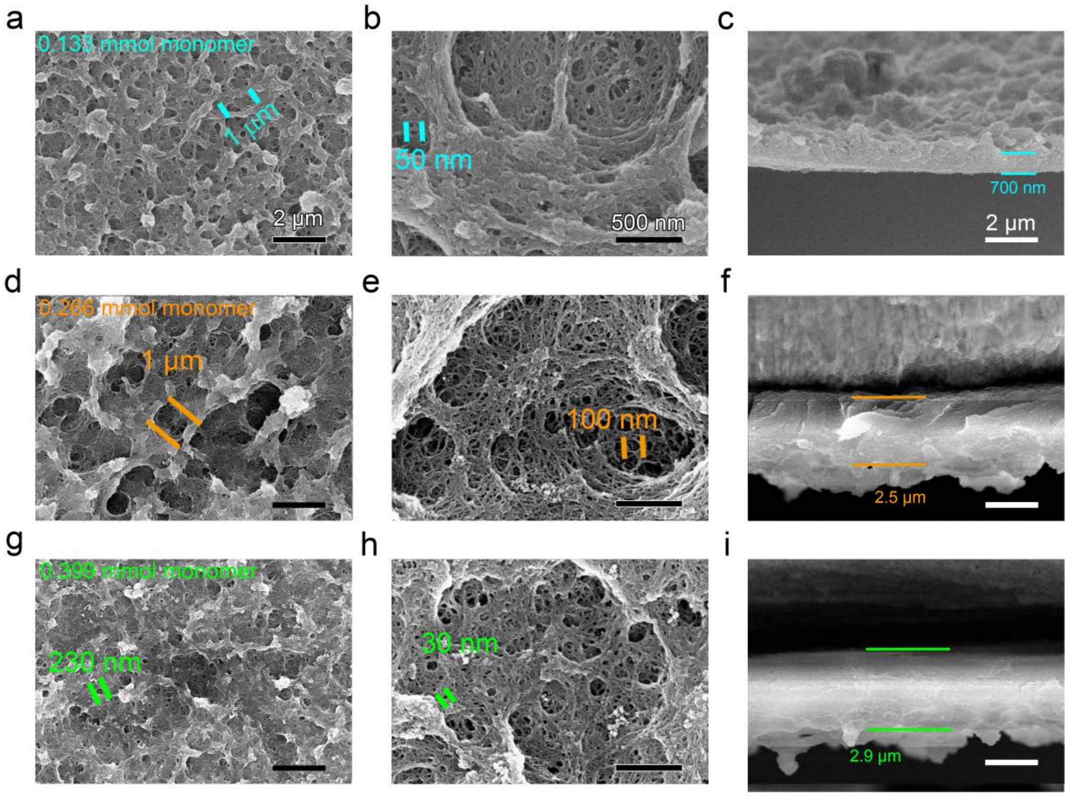

Supplementary Figure 9. The morphology of the HsGDY film synthesized by different amount of monomer. (a–c) 0.133 mmol, (d–f) 0.266 mmol, (g–i) 0.399 mmol. Scale bar, 2 μm (**a**, **c**, **d**, **f**, **g**, **i**), 500 nm (**b**, **e**, **h**).

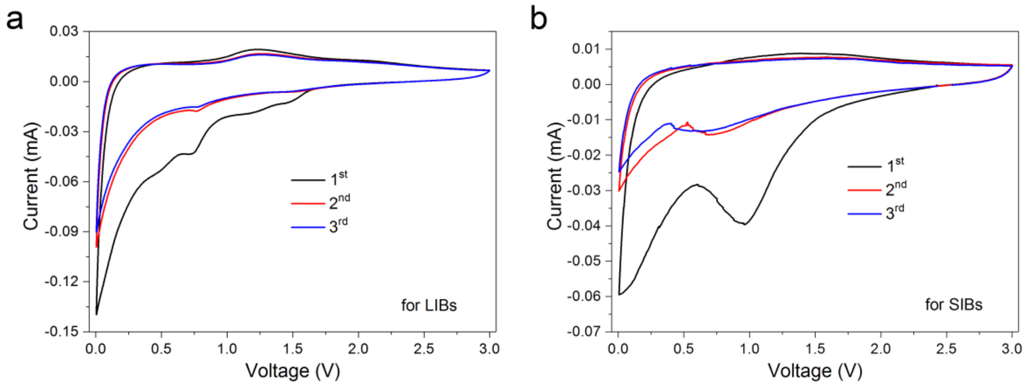

Supplementary Figure 10. CVs of the HsGDY electrodes at a scanning rate of 0.1 mV s^{-1} during the initial three cycles.

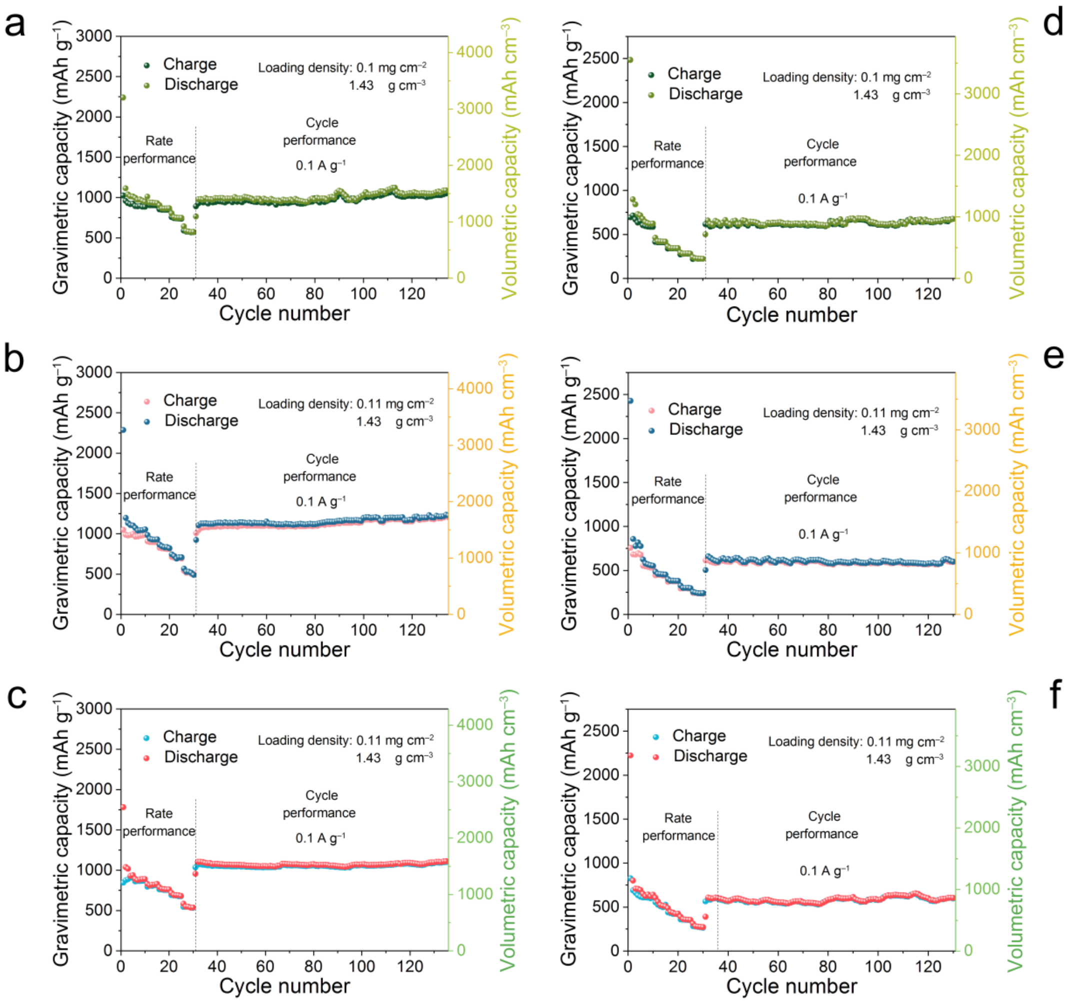

Supplementary Figure 11. The reproducibility of the electrochemical performance for HsGDY electrodes, (a–c) in LIBs, (d–f) in SIBs.

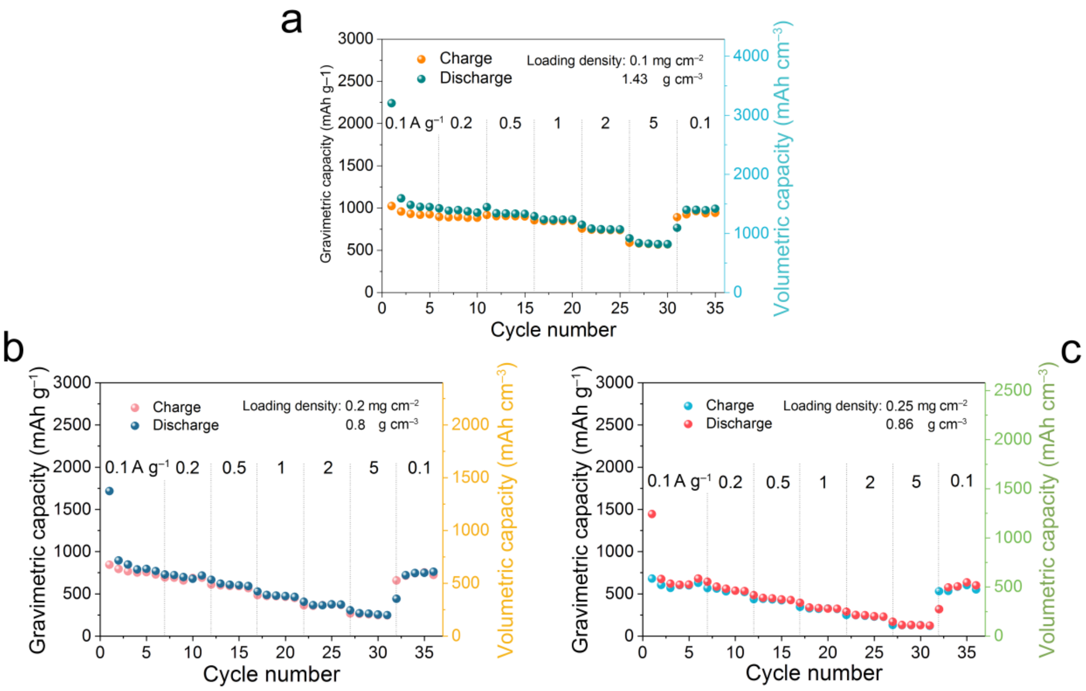

Supplementary Figure 12. Rate performance of the HsGDY electrodes synthesized by different amount of monomer in LIBs, (a) 0.133 mmol, (b) 0.266 mmol, (c) 0.399 mmol.

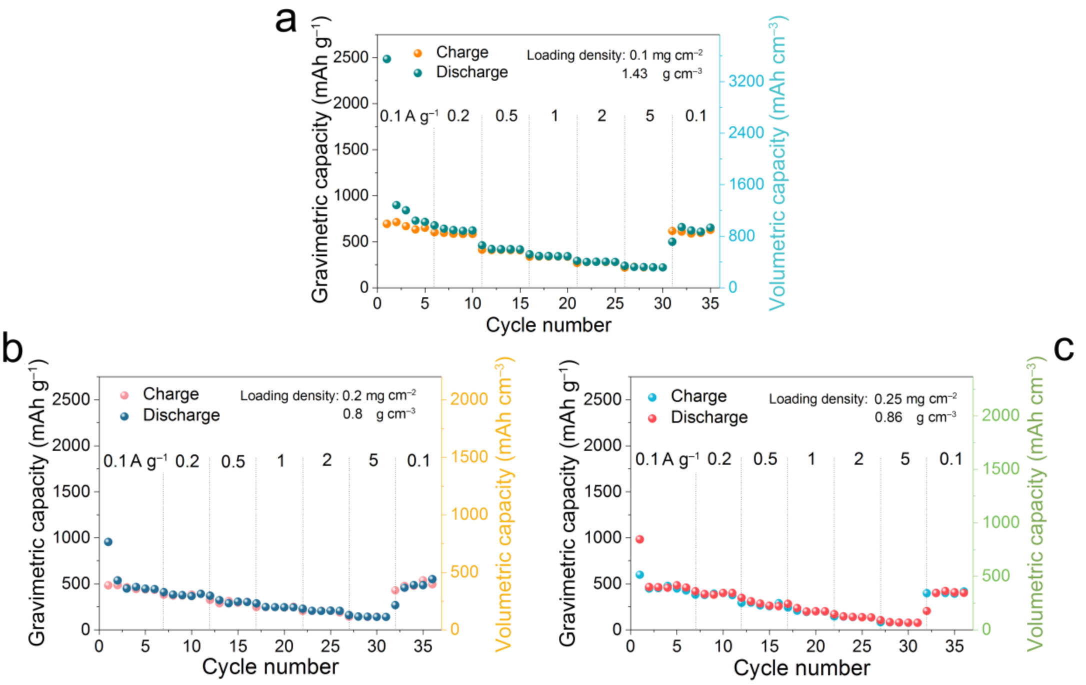

Supplementary Figure 13. Rate performance of the HsGDY electrodes synthesized by different amount of monomer in SIBs, (a) 0.133 mmol, (b) 0.266 mmol, (c) 0.399 mmol.

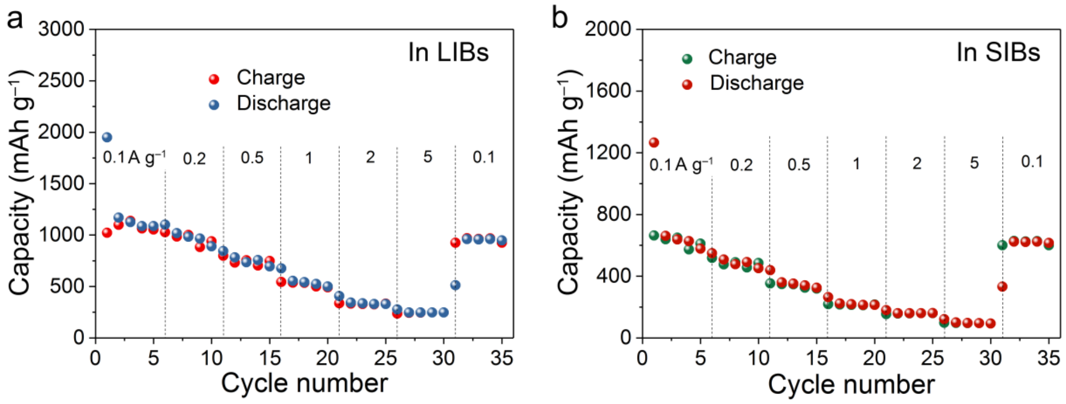

Supplementary Figure 14. Rate performance of the HsGDY electrodes coated on copper foil, (a) in LIBs, (b) in SIBs.

a b

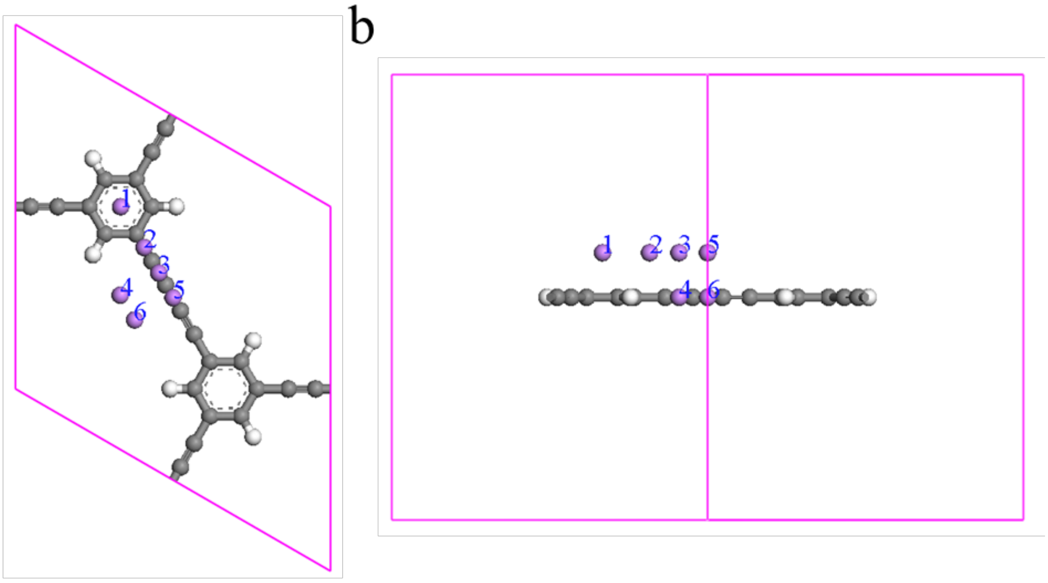

c

	$E_b(1)$/eV	$E_b(2)$/eV	$E_b(3)$/eV	$E_b(4)$/eV	$E_b(5)$/eV	$E_b(6)$/eV
Li	1.11	0.96	1.03	0.74	1.01	0.74
Na	0.73	0.67	0.64	0.51	0.69	0.53

$E_b(n)$ represents the binding energy of single Li atom at different sites.

Supplementary Figure 15. The calculated binding energies of single Li/Na atom at different sites on selected repeating unit. (a) top view and (b) cross-section view of Li/Na-$C_{24}H_6$ complex and (c) its binding energy (E_b). Here $E_b = (E_{HsGDY} + nE_{Li/Na} - E_{complex})/n$, where E_{HsGDY}, $E_{Li/Na}$, $E_{complex}$ are the energies of HsGDY ($C_{24}H_6$), single Li or Na atom, and *n*Li(or Na)/HsGDY complex, respectively; *n* is the number of adsorbed Li or Na atoms.

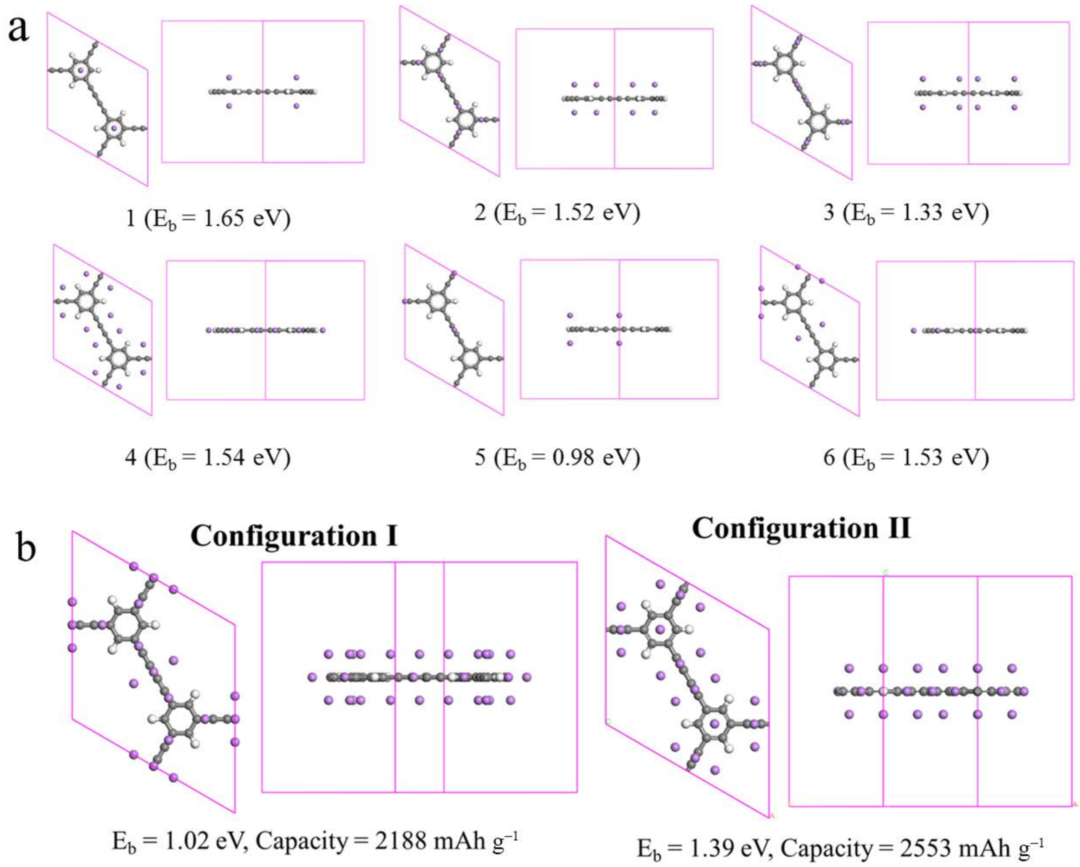

Supplementary Figure 16. The calculated binding energies of Li atom on HsGDY. (a) Multiple equivalent Li atoms at one site on selected repeating unit. (b) Representative adsorption structures and corresponding binding energies (E_b) and storage capacity for Li atoms on HsGDY. $E_b = (E_{HsGDY} + nE_{Li} - E_{complex})/n$, where E_{HsGDY}, E_{Li}, $E_{complex}$ are the energies of HsGDY ($C_{24}H_6$), single Li atom, and *n*Li/HsGDY complex, respectively; *n* is the number of adsorbed Li atoms. Capacity = *n*F/3.6M, where F = 96500 mAh and M is the mass of HsGDY ($C_{24}H_6$) in the unit of gram.

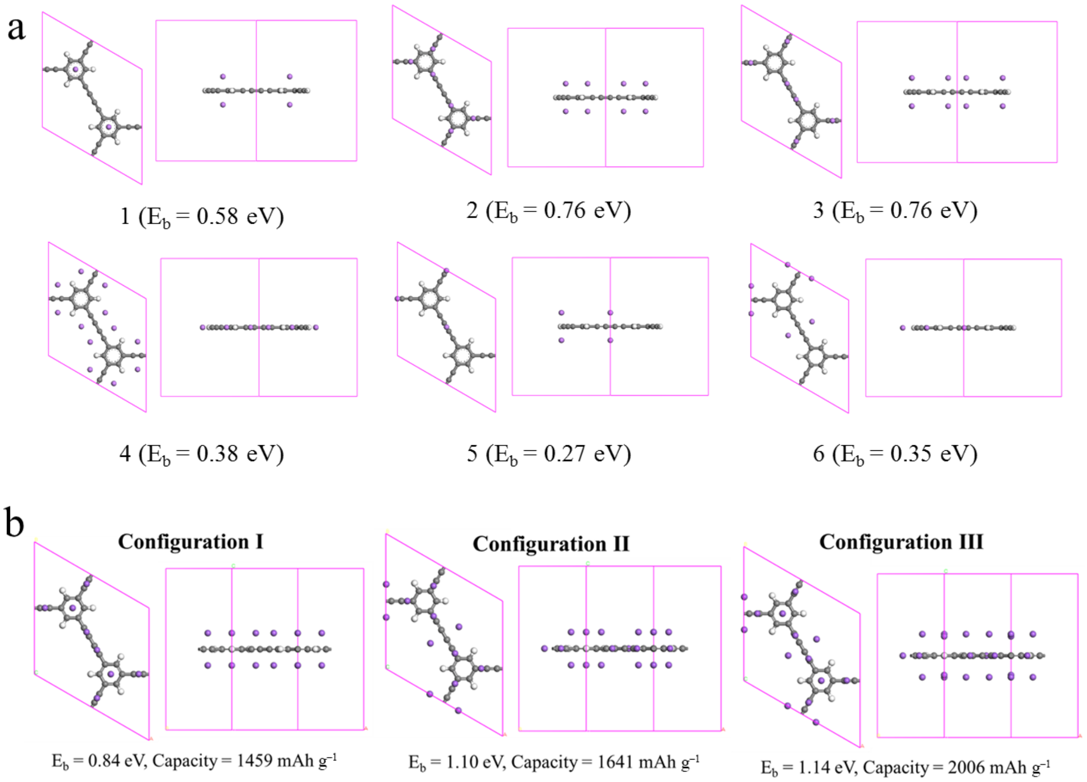

Supplementary Figure 17. The calculated binding energies of N atom on HsGDY. (a) Multiple equivalent Na atoms at one site on selected repeating unit. (b) Representative adsorption structures and corresponding binding energies (E_b) and storage capacity for Na atoms on HsGDY. $E_b = (E_{HsGDY} + nE_{Na} – E_{complex})/n$, where E_{HsGDY}, E_{Na}, $E_{complex}$ are the energies of HsGDY ($C_{24}H_6$), single Na atom, and *n*Na/HsGDY complex, respectively; *n* is the number of adsorbed Na atoms. Capacity = *n*F/3.6M, where F = 96500 mAh and M is the mass of HsGDY ($C_{24}H_6$) in the unit of gram.

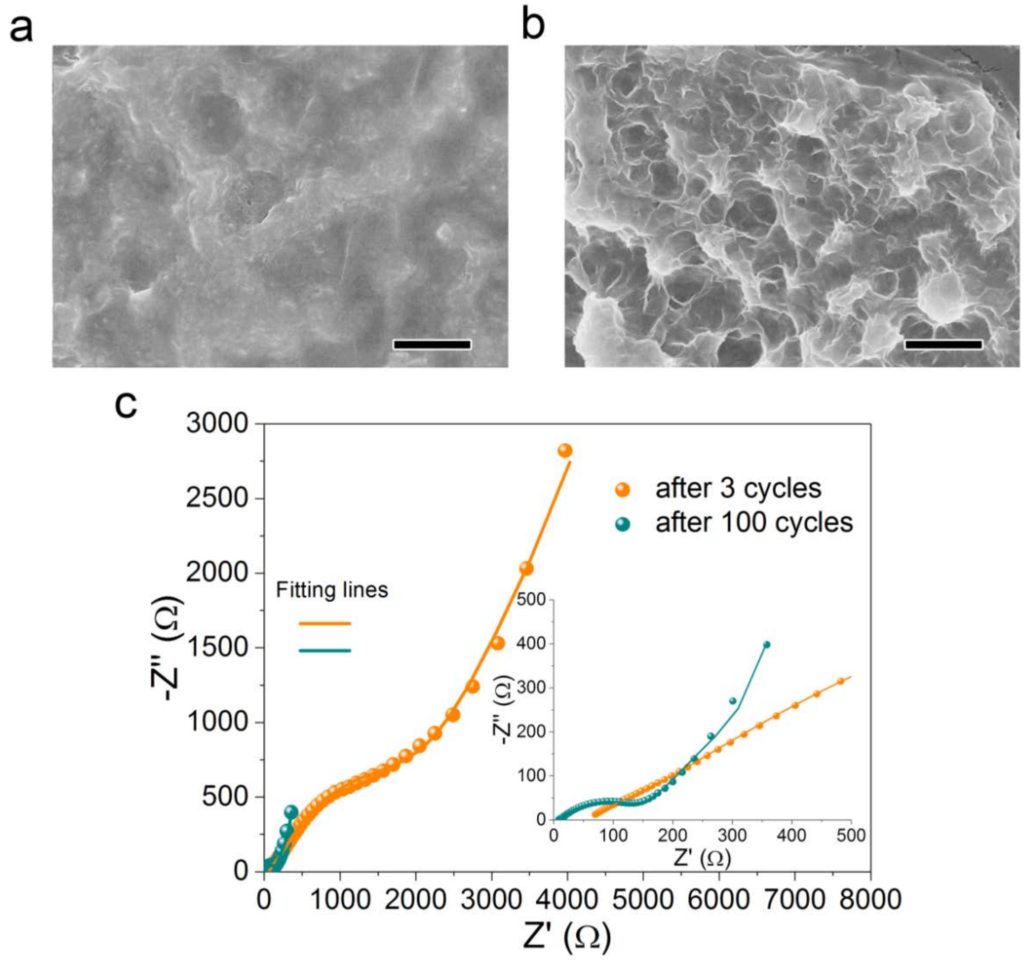

Supplementary Figure 18. The characterization of the electrode after cycles. The SEM images of the electrode for (a) LIBs and (b) SIBs. (c) The EIS spectra of the electrodes for LIBs. All scale bars are 2 µm.

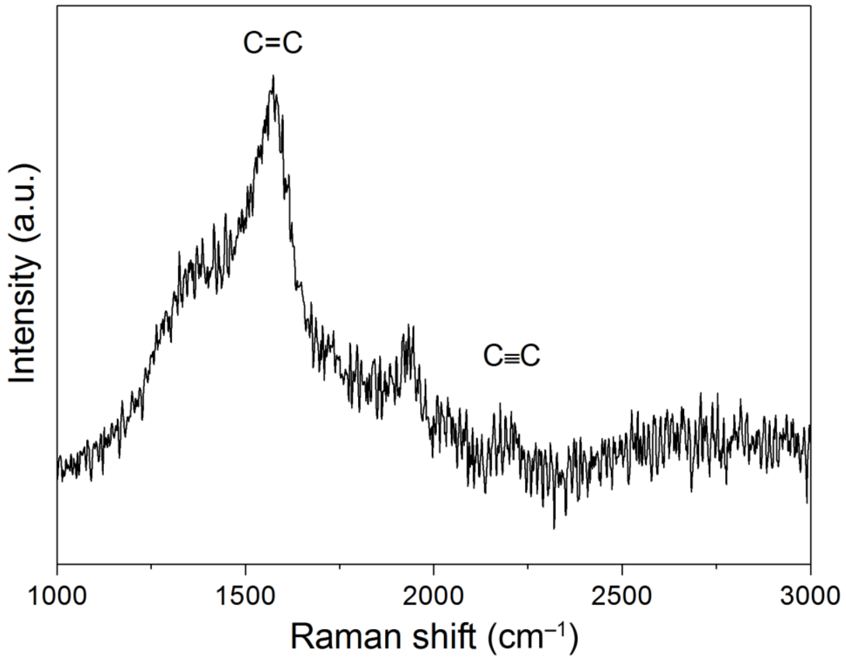

Supplementary Figure 19. Raman spectrum of HsGDY after cycles for LIBs.

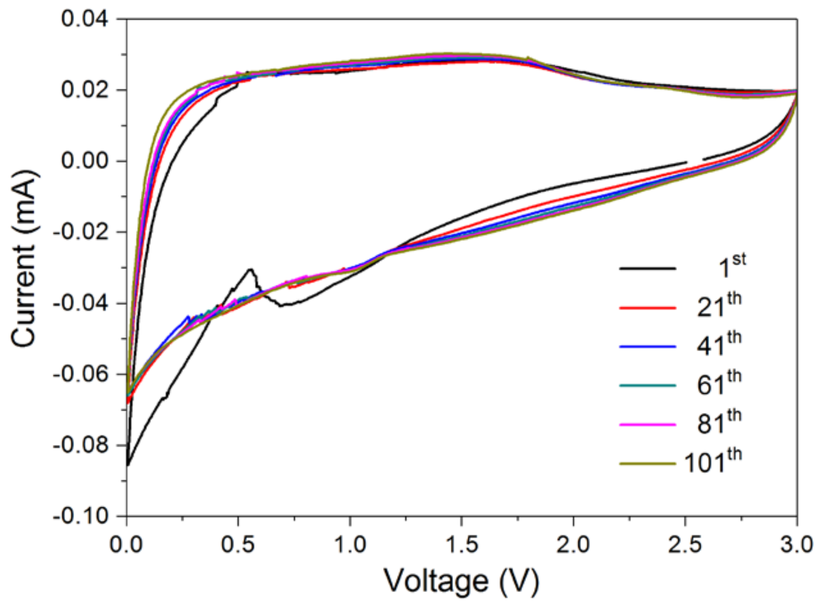

Supplementary Figure 20. CVs of the HsGDY electrode after different cycles for SIBs at a scanning rate of 0.5 mV s^{-1}.

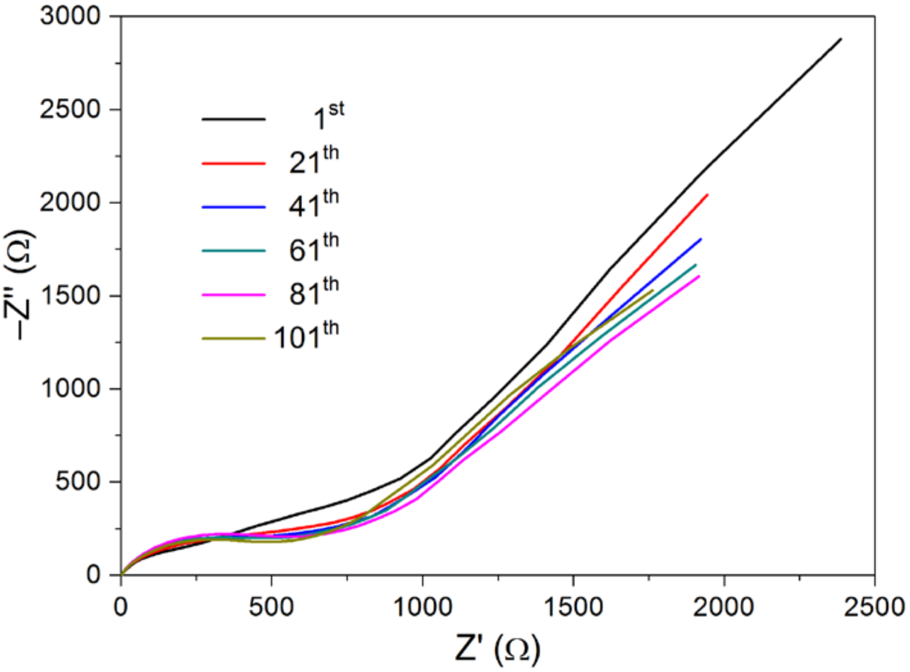

Supplementary Figure 21. EIS spectra of the HsGDY electrode after different cycles for SIBs.

Supplementary Table 1. Kinetic parameters of the electrodes during different cycles.

Samples	R_s (Ω)	R_{ct} (Ω)
HsGDY electrode after 3 cycles	109.8	1897.0
HsGDY electrode after 100 cycles	4.0	131.7

Supplementary Notes

The corresponding ^{1}H NMR, ^{13}C NMR spectra are shown in Supplementary Figure 1 and 2, indicating high purity of the monomer. (The peaks at the range of 0–2 ppm in ^{1}H spectrum and 0–40 ppm in ^{13}C NMR spectrum were originated from the TBAF and dissociative protected groups from the monomer). ^{1}H and ^{13}C NMR spectra were recorded on a Bruker AVANCE–III 600 MHz spectrometer. All chemical shifts of ^{1}H were referenced to tetramethylsilane $CDCl_3$ (δ = 7.29 ppm) and ^{13}C NMR chemical shifts were referenced to $CDCl_3$ (δ =77.00 ppm).

The DFT calculation method is provided in experimental section at the beginning of the supplementary information. Before constructing the representative adsorption structures of Li/Na on HsGDY in Supplementary Figures 16–17b, six possible sites for the storage of single Li/Na atom are selected and confirmed by the appropriate binding energy (Supplementary Figure 15).

Furthermore, the binding energies of multiple equivalent Li/Na atoms on six different positions of HsGDY are calculated (Supplementary Figure 16–17a). The result indicates the symmetrical configuration is benefit for the stability. However, it is impossible to fill all the above possible storage sites at the same time because of the repulsion interaction between Li/Na atoms in nearest-neighbor sites. Following the above principles, and guaranteeing the most stable binding energies and maximization of Li storage, a stable Li_{28}–$C_{24}H_6$ complex (configuration II) is selected as the optimized configuration. The binding energy for per Li atom in this configuration is calculated to be 1.39 eV, which is even more stable than that of single type of Li atoms adsorbed configuration (Supplementary Figure 15). The result demonstrates the configuration is reasonable. Furthermore, the storage capacity is calculated to be 2553 mAh g^{-1} by means of the Li_{28}–$C_{24}H_6$ configuration. The same strategy is applied in the calculation of Na storage. However, Na displays stronger repulsion and larger atomic diameter than those of Li, which lead that the number of Na which can be put in the storage sites is less than that of Li. In consequence, a stable Na_{22}–$C_{24}H_6$ (configuration III) is obtained as shown in Supplementary Figure 17.

Supplementary Methods

Reagent. Triethylamine (TEA) and pyridine were pretreated under reflux with KOH. Tetrahydrofuran (THF) was pretreated under reflux with sodium crumbs. Cu foil was purchased from Alfa Aesar and treated in 1M HCl, water, ethanol and acetone for 10 minutes, respectively, dried under a flow of nitrogen and used immediately. Tribromobenzene, Trans-dichloro-bis(triphenyl-phosphine) Palladium(II) ($Pd(PPh_3)_2Cl_2$), (Trimethylsilyl)acetylene ($(CH_3)_3SiC{\equiv}CH$), Copper (I) iodide (CuI), Triphenyl phosphorous (PPh_3), tetrabutylamonium fluride (TBAF) were purchased from alladin and used without further purification.
